# Selective Age Effects on Visual Attention and Motor Attention during a Cued Saccade Task

**DOI:** 10.1155/2014/860493

**Published:** 2014-05-12

**Authors:** Wendy E. Huddleston, Brad E. Ernest, Kevin G. Keenan

**Affiliations:** Department of Kinesiology, University of Wisconsin-Milwaukee, PT-PAV 350, P.O. Box 413, Milwaukee, WI 53201-0413, USA

## Abstract

*Objective*. Visual information is often used to guide purposeful movement. However, older adults have impaired responses to visual information, leading to increased risk for injuries and potential loss of independence. We evaluated distinct visual and motor attention contributions to a cued saccade task to determine the extent to which aging selectively affects these processes. *Methods*. Nineteen healthy young (18–28 years) and 20 older (60–90 years) participants performed a cued saccade task under two conditions. We challenged motor attention by changing the number of possible saccade targets (1 or 6). *Results*. Older adults had difficulty in inhibiting unwanted eye movements and had greater eye movement inaccuracy in the hard condition when compared to the younger adults and to the easy condition. Also, an inverse relation existed between performance on the visual and motor components of the task in older adults, unlike younger adults. *Conclusions*. Older adults demonstrated difficulty in both inhibiting irrelevant saccade targets and selecting correct saccade endpoints during more complex tasks. The shift in relations among attention measures between the younger and older participants may indicate a need to prioritize attentional resources with age. These changes may impact an older adult's ability to function in complex environments.

## 1. Introduction

Most people depend heavily on visual cues to guide purposeful movement within the environment. Some older adults respond more slowly than younger adults to changes in the environment [1–4], negatively affecting functional independence and leading to increased risk of injuries related to traffic accidents or falls [5, 6]. These age-related declines in response to the environment may be due to changes in cognitive processes such as attention. Attentional processes contribute to each stage of a visually guided movement, particularly the selection of visual information relevant to the current task [7–9] and the selection of the correct motor plan to successfully perform the action [10–13]. The component, or components, of a visually guided task at which the effects of age-related declines in attention most significantly alter function [2, 14–16], is under debate. For example, deficits in both visual processing [17, 18] and motor control [2, 19] have been reported in older adults. Separate basic tests of visual perception (line length discrimination) as well as motor function (timed finger tapping) illustrate modality-specific declines in older adults [2].

Performance declines in older adults are oftentimes attributed to decreased processing speeds [20, 21]. The Processing-Speed Theory posits that cognitive acts are more difficult for older adults because it simply takes longer to perform all aspects of the task. This increase in time required for processing leads to diminished performance because either the activity is time-limited, or the increased time for processing each component in serial tasks prevents information from early steps still being available for later steps. However, increases in reaction time associated with both the visual and motor systems together do not fully explain deficits noted in a choice reaction time task [2]. Attentional deficits may also contribute to slowing of responses. Thus, in addition to age-related generalized slowing [21, 22], specific declines in executive function, such as attention, may subsequently explain observed declines in the control of eye movements [23, 24] in complex situations (but not in simple scenarios; see [19, 25]).

Other theories of cognitive aging may further explain declines in attentional processes involved in visuomotor tasks. Some older adults have difficulties processing task conditions or requirements as described by the Task Context Theory [26, 27]. Context, in this case, is described as relevant information to the task at hand such as the goal of the movement or specific instructions. Top-down, or voluntary, attentional processes require contextual information to correctly select information required for successful completion of the task. Impaired maintenance of context due to age may lead to an inability to correctly or efficiently select information necessary for successful and timely completion of the task, such as to move the eyes to a correct location at the correct time [23, 24]. The complementary view to older adults having difficulty selecting relevant task information is the inability to inhibit irrelevant information (Inhibitory Deficit Theory). Older adults tend to have difficulty suppressing information irrelevant to the current task [28, 29], which then leads to cognitive decline. Inhibition of irrelevant information is critical for controlling access to the focus of attention, deleting irrelevant information from attention and working memory stores, and suppressing or restraining planned, but inappropriate, responses [29]. Although the physiological mechanisms behind the two theories differ, behavioral predictions from both are typically similar.

Much evidence suggests that older adults have difficulty making the correct selection of task-related information and/or suppressing all unnecessary information [14, 17–19, 30–33]. For example, older adults demonstrate greater slowing in reaction times with an increase in the number of choices for reach targets in a visual pop-out task [34] and have failed to follow precues regarding which effector (left or right hand) to use [35]. Also, when healthy older participants are asked to specifically attend to a salient visual cue to guide movement, either error rates increase compared to a simple reaction time task [36] or movement speed decreases compared to younger adults [37]. However, in these studies the visual cue to guide movement “pops out” via a rapid onset or a significant difference in cue appearance. Visual cues that significantly differ in appearance or onset typically have a strong involuntary attention saliency signal. One question remaining unanswered is how older adults respond to changes in the complexity of the motor task when the target of action is no more visually salient than distractors, minimizing any involuntary capture of attention. In complex environments, such as driving, the ability to voluntarily select visual information and initiate correct movements is critical for successful goal-directed actions. The correct selection of a motor plan, via attention-mediated mechanisms, may play a critical role in preventing driving accidents, which might in turn increase functional independence in older adults. In this case, top-down attentional factors related to task context determine the salience of the desired movement. A task in which the targets are no more salient than the distractors may have more ecological validity related to older adults functioning within the home, driving, or avoiding falls in the community.

It is critical to understand the nature of the decline in endogenous visually guided movements in older adults so as to develop approaches to maximize functional independence within this population. Thus, the purpose of the current study was to explicitly evaluate visual and motor domains of attention contributing to a cued saccade visuomotor task to identify which attentional components show an interaction effect between age and motor attentional load. In younger adults, increasing the attentional load during a cued saccade task in either the visual or motor domain negatively impacted performance within that specific domain without affecting the opposite one, indicating a functional dissociation of the two processes in this age group [38]. The unique aspect of the present experiment is the use of measures to separately assess different attention domains in older adults.

As in our previous study [38], participants performed saccades to one of six peripheral targets based on a centrally located cue. Younger and older adults performed the task with two levels of motor attention load under vigorous temporal constraints, all while maintaining constant visual perceptual task demands across conditions (six cue letters were used in both motor load conditions). We increased the motor attention load by changing the number of possible saccade targets from 1 to 6. In the hard condition (i.e., 6 possible saccade targets), participants had to select the correct target and inhibit other incorrect targets in a time-sensitive manner (six cue letters mapped to six different targets). In the easy condition, participants only performed a saccade to one location regardless of the visual cue and thus did not have to inhibit selection of incorrect targets (six cue letters mapped to one common target) yet still would have to select the single correct targets. We presented the cue letters at a fast rate (4 per second) to press the temporal constraints of the task. Using this paradigm, we tested the hypothesis that older adults would have greater difficulty than younger adults when more irrelevant information (more choices in saccade targets) was available during the hard condition. We could explicitly test predictions made by three theories of cognitive aging (Task Context Theory, Inhibitory Deficit Theory, and Processing-Speed Theory) by evaluating several dependent measures including visual attention errors (incorrectly perceiving the cue letter either by missing a cue or by perceiving one when one was not shown), saccade target selection errors (initially performing saccades to an incorrect target and then self-correcting), saccade endpoint accuracy and variability, and reaction time. All three theories of cognitive aging would predict an age effect for visual attention errors, with older adults performing worse on that component of the task. However, we did not anticipate any condition effect in visual attention errors as we did not manipulate visual attention load across conditions, and due to our previous findings in younger adults [38]. We also predicted that older adults would respond more slowly (based on the Processing-Speed and the Task Context Theories) and make more saccade target selection errors, and these age differences would increase in the hard condition (based on the Task Context and Inhibitory Deficit Theories). Our last prediction was that older adults would be as accurate and reliable as younger adults in saccade endpoint accuracy and variability in the easy condition, but not in the hard condition as predicted by the Task Context Theory and the Inhibitory Deficit Theory. Preliminary data have been previously presented elsewhere [39].

## 2. Materials and Methods

Younger and older participants performed a cued saccade task to identify the effects of task difficulty and age on visual and motor attentional abilities. For the current study, we define visual attention as the selection of task-relevant cue letters from all distractors, a definition consistent with others [7–9]. Motor attention has been previously described as involving the selection of movement target, trajectory, and effector [40]. Because effector selection (the eyes) was determined by the task and did not change over the course of the experiment, we define motor attention as the selection of the correct saccade target and associated accuracy [10–13, 41]. In this experiment we challenged motor attention by changing the number of possible saccade targets between the easy and hard task conditions. We were particularly interested in potential age-related changes in performance when motor attention was challenged in the hard task condition.

### 2.1. Participants

Twenty healthy older adults (60–90 years, 9 females, 4 left-handed) and 20 healthy young adults (18–28 years, 11 females, 4 left-handed), with normal or corrected-to-normal vision, completed the study. One younger adult could not achieve greater than 50% accuracy during training on the cued saccade task and so all data from this participant were removed from analysis. All participants provided written informed consent as approved by the University of Wisconsin-Milwaukee Institutional Review Board. Exclusion criteria included an inability to sit comfortably for extended periods, self-report of diseases of the eye, and neurological or musculoskeletal conditions that would affect task performance. All participants lived independently within the community.

### 2.2. Equipment

We recorded eye movement with an infrared R6 Remote Optics Eye Tracking System (Applied Science Laboratories, Bedford, MA) at 120 Hz. Eye tracking data were collected with a personal computer (DELL, Austin, TX). Participants completed a full-field 9-point eye tracking calibration routine prior to testing.

### 2.3. Stimulus and Task

**Figure 1:**
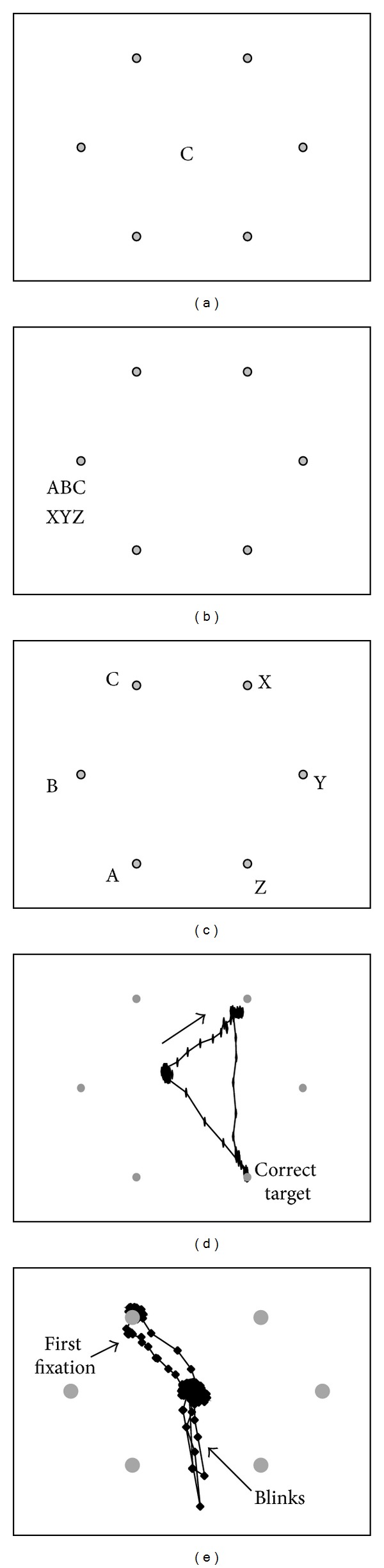
Stimulus and task. (a) The stimulus as seen by the participant. The central letter (“C” in this figure) changed every 250 ms. (b) An example of stimulus-response mapping for the easy condition. In this case, the participant would perform a saccade to the middle left location whenever any of the 6 target letters were shown. The mapping was not present during the data collection and the mapping changed for every set of 12 trials. (c) An example of stimulus-response mapping for the hard condition in which each cue letter was mapped to a unique peripheral target. (d) An example of a participant making a target selection error. The participant initially selected an incorrect target (top right) and then made a correction to the correct bottom right location. In this case the participant correctly identified the cue letter (“Z”) and recalled the correct mapping (bottom right) but chose an initially incorrect target. (e) A correct trial illustrating saccadic eye movement. In this case, the participant made an initial saccade in the correct direction and then further corrected to the target location prior to returning gaze to the central letter stream. During this trial, the participant overshot in the horizontal direction and undershot in the vertical direction during the initial saccade.

The stimulus and task have been described previously [38, 42]. We created and presented the visual stimuli using Presentation software (Neurobehavioral Systems, Albany, NY). The stimulus (Figure 1) consisted of six peripheral targets (13° eccentricity). In the center of the monitor, a rapid serial visual presentation (RSVP) of random letters (subtending 1.2 × 1.8° visual angle) occurred at 4 Hz (marked by “C” in Figure 1(a)). Participants performed saccades as quickly and as accurately as possible to one of six peripheral target locations based on cue presentation of “A,” “B,” “C,” “X,” “Y,” or “Z” in the RSVP stream of letters. For example, if “B” was presented, the participant was instructed to look as quickly and as accurately at a predetermined peripheral target location and then focus his or her attention (and gaze) back to the RSVP stream of letters to await the next trial. Participants were instructed to make corrective saccades until they looked at the correct target. If a target letter was not presented, participants continued to focus centrally on the RSVP stream of letters. Target letters were randomly presented no closer than six seconds apart within each set. The duration between cue letter presentations was considered to be a single trial. Each cue letter was presented 2 times, for a total of 12 trials per set. Distractor letters consisting of the remaining letters of the alphabet were shown between target letter presentations. Prior to each set of 12 trials, each participant was shown a mapping between cue letter and target location (Figures 1(b) and 1(c)). This mapping changed for every set of trials and differed between the easy and hard task conditions. An “easy” mapping cued the participant to perform a saccade to a single location when any of the six cue letters were presented (Figure 1(b)). A “hard” mapping instructed the participant to perform a saccade to a different location for each of the cue letters presented (Figure 1(c)). By changing the number of possible saccade targets from one to six between conditions, we manipulated motor attention load. It is important to note that visual attention load remained constant between the two task conditions as the number of target cues was always six, and the six peripheral saccade targets remained on the screen (even when no cue letter was mapped to them in the easy condition). Participants were able to study the mapping for as long as they wished and were tested to confirm knowledge of the correct target location mapping prior to starting each set of trials. Participants completed three sets of 12 trials for each condition. Prior to collecting experimental data, participants trained on the task until they were able to achieve 75% of letter identification (1–3 sets of 12 trials for all participants).

### 2.4. Data Analysis

Eye tracking data collected during the cued saccade task were segmented into individual trials using custom software (BloomTech, Richfield, WI) written in LabVIEW (National Instruments, Austin, TX). Each trial was segmented into the central fixation, the first saccade, and the first fixation for further analysis. Data were collapsed across target locations within each condition. Trials in which eye movement data were lost or in which blinks occurred during the first saccade or subsequent fixation were removed from analysis.

Dependent variables assessed visual or motor attention. The dependent measure for the visual attention domain included the visual attention error rate. We calculated the visual attention error rate by adding the false positive rate and the miss rate together. We summed the values from these two types of errors, as the denominators were slightly different for each rate as described below. We determined a false positive occurred when a participant performed a saccade to a minimum of 75% of the distance to any peripheral target without cue letter having been presented within 1500 ms prior to the saccade. The false positive rate was calculated by dividing the number of false positives by the total number of trials plus the number of false positives. The false positive rate was calculated in the manner described above because a false positive essentially added a trial to the participant's experience. To not account for the false positives in the denominator of the equation would have falsely inflated the error rate. The miss rate was calculated by dividing the number of trials in which participants missed target letters (participant did not perform a saccade within 3000 ms of cue letter presentation) by the total number of trials. Conversely, we did not take into account the false positives when calculating the miss rate because then the score would have been artificially deflated. Twelve target letters were presented in a single run and we wanted the miss rate to represent the percentage of target letters missed.

Motor attention dependent measures included the target selection error rate, reaction time, first saccade endpoint accuracy relative to target, and within-participant saccade endpoint variability. Target selection error rates, indicating faulty motor planning, were calculated based on the number of times participants initially performed a saccade to an incorrect target but then made a correction to the appropriate location (Figure 1(d)). When making target selection errors, participants correctly identified the cue letter but initially selected the incorrect target. In this case, the perceptual portion of the task was done correctly, but the early stages of motor planning were executed incorrectly. It is important to note that these errors are not an issue of working memory, as the participant was able to ultimately perform a saccade to the correct location. When calculating the target selection error rate, the number of trials in which the initial saccade target selection was incorrect was divided by the total number of trials in which the perceptual portion of the task was performed correctly. Reaction time and saccade endpoint accuracy were calculated based on the first saccade endpoint after the cue was presented (Figure 1(e)). Reaction time was calculated separately for correct trials and for trials in which a trajectory selection error occurred. Saccade endpoint accuracy and variability were only calculated for correct trials. Saccade endpoint accuracy is primarily dependent on visual perception of the saccade target and motor planning [43]. Sensory input regarding target location was stable throughout the current experiment as the saccade targets were present at all times during the experiment; thus we considered saccade endpoint accuracy to reflect motor planning. Saccade endpoint accuracy was calculated for the horizontal and vertical directions separately in degrees of visual angle by computing the horizontal and vertical distance between the center of mass of the first endpoint and the coordinates of the correct target location. Overshoot for all of the targets was considered a positive error, and undershoot was considered negative. This analysis allowed us to quantify the direction of the error. Additionally, we calculated an absolute error vector to quantify the magnitude of saccade endpoint accuracy. We calculated the root mean square distance (in degrees of visual angle) between the first fixation and the actual target location using the horizontal and vertical error values described above. We also investigated the within-participant variance of saccade endpoint accuracy by taking the within-participant standard deviation of the accuracy. Small standard deviations would be interpreted as consistent motor planning.

A mixed-model 2 × 2 repeated measures ANOVA was used to identify condition (within participants) and age (between participants) differences, along with possible interactions for all dependent measures. Where effect size could be calculated, either a partial eta-squared (*h*_*p*_^2^) or a Cohen's *d*-value is reported in the results. Statistical analysis was performed using SPSS 19.0 (SPSS Inc, Chicago, IL). The level of significance for all statistical tests was set at 0.05.

## 3. Results

All dependent measures demonstrated significant age effects for the easy and hard conditions. Means and standard deviations for all measures from both younger and older participants and across conditions are presented in Table 1.

### 3.1. Motor Attention Domain

Significant age effects were observed for target selection error rates, reaction time, saccade endpoint accuracy, and saccade endpoint variability.

#### 3.1.1. Target Selection Errors

**Figure 2:**
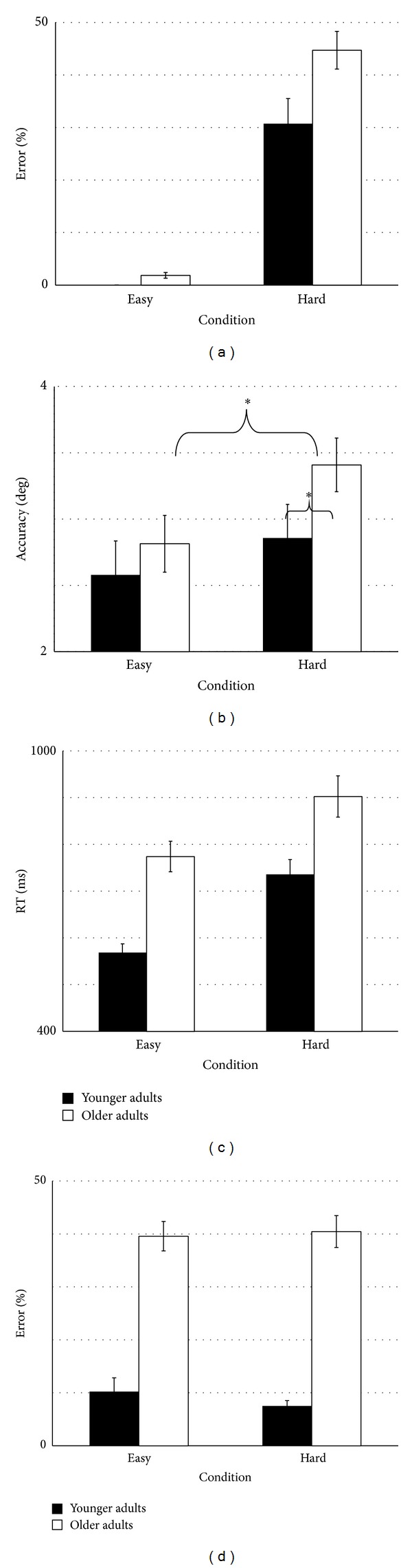
Effects of task condition and age on performance. (a) Both younger and older participants made more target selection errors in the hard condition compared to the easy condition. The older participants also made more target selection errors than their younger counterparts. (b) Mean magnitude of the saccade endpoint accuracy error relative to the target location. (c) Reaction time. Both main effects of condition and age were significantly different with no significant interaction present. (d) Older participants made significantly more visual perception errors (misses and false positives) than the younger participants in both conditions (represented along *x*-axis). All data are presented as mean ± SEM.

We had hypothesized that if older adults had difficulty suppressing the irrelevant saccade targets, the largest effects of task condition would be seen in the target selection error rate and not in measures of saccade endpoint accuracy or reaction time. Participants in both age groups did not make many target selection errors when only one saccade direction was required for the task (easy condition), although even in this condition older adults made slightly more errors (2%). However, older adults made 14% more target selection errors than their younger counterparts when the number of possible saccade targets increased between conditions (condition main effect *F*(1,37) = 162.980, *P* < 0.001, *h*_*p*_^2^ = 0.815; age main effect *F*(1,37) = 7.361, *P* = 0.010, *h*_*p*_^2^ = 0.166; condition × age interaction *F*(1,37) = 4.433, *P* = 0.042, *h*_*p*_^2^ = 0.107; Figure 2(a)). This increase in the initial target selection error rate in older adults was not related to working memory as participants did ultimately perform a saccade to the correct location even though peripheral letters were not present during data collection (Figure 1(d)). These results are consistent with older adults having more difficulty suppressing irrelevant saccade targets, and/or more difficulty determining task context, after the visual cue was identified than the younger participants.

#### 3.1.2. Saccade Endpoint Accuracy

We also wanted to determine the level of difficulty older adults had with the selection of the correct saccade path. We predicted that older adults would be as accurate and consistent as younger adults in selecting saccade targets in the easy condition, but not in the hard condition. When evaluating the overall magnitude of the error of the first saccade endpoint, all participants made greater errors in the hard condition than the easy condition (*F*(1,37) = 5.459, *P* = 0.025, *h*_*p*_^2^ = 0.129; Figure 2(b)). Interestingly, the main effect of age was not significant (*F*(1,37) = 2.310, *P* = 0.137, *h*_*p*_^2^ = 0.059; age × condition interaction *F*(1,37) = 0.488, *P* = 0.717, *h*_*p*_^2^ = 0.019). However, post hoc analysis showed that the effect of task difficulty on saccade endpoint accuracy was driven by significantly worse saccade endpoint accuracy by the older adults in the hard condition compared to the easy condition (one-tailed paired *t*-test *t*(19) = 1.939, *P* = 0.034, *d* = 0.720) and worse accuracy of the older adults compared to the younger adults in the hard condition (one-tailed *t*-test *t*(37) = 1.754, *P* = 0.044, *d* = 0.560). Older adult accuracy in the easy condition was no different than the performance of the younger adults, showing that the mechanics of performing the saccade did not contribute to the change in accuracy in the hard condition. Also, we attribute changes in accuracy between task conditions in older adults to changes in motor attention rather than visual attention as visual attention load did not change. Although older adults were less accurate in the hard condition, they were no more variable in their saccade trajectories when compared to younger participants (*F*(1,37) = 0.049, *P* = 0.825, *h*_*p*_^2^ = 0.001). Also, no effect of task difficulty was present in saccade endpoint variability (*F*(1,37) = 0.763, *P* = 0.388, *h*_*p*_^2^ = 0.020; age × condition interaction *F*(1,37) = 0.071, *P* = 0.792, *h*_*p*_^2^ = 0.002).

#### 3.1.3. Reaction Time

In this task, only the number of possible saccade targets changed between task conditions; thus changes in reaction time (the time between cue presentation and the initiation of eye movement) would presumably be due to motor planning differences. We predicted that older adults would respond more slowly, and these age differences would be enhanced in the hard condition. In both age groups, reaction times for correct trials were significantly longer in the hard condition (*F*(1,37) = 38.201, *P* < 0.001, *h*_*p*_^2^ = 0.508; Figure 2(c)), with older adults being significantly slower than the younger adults in both the hard and easy conditions (*F*(1,37) = 22.706, *P* < 0.001, *h*_*p*_^2^ = 0.380; age × condition interaction *F*(1,37) = 0.655, *P* = 0.423, *h*_*p*_^2^ = 0.017).

We then calculated within-participant reaction time differences between the easy and hard conditions. In the easy task condition, participants knew the correct saccade location without having to identify specific letter cues (e.g., “A” versus “B”). The participant would simply make the saccade when any target letter was identified. Thus, the reaction time in this scenario mostly reflects the time for visual perception and saccade execution. In the hard condition, the correct target letter had to be specifically identified (i.e., not only that a target letter was shown, but rather which target letter was shown), the correct mapping applied, and the correct target selected. The longer reaction time in the hard condition reflects the additional components to the visuomotor task. If true, subtracting the easy condition reaction time from the hard condition reaction time would provide an index of the temporal cost for the additional steps. No significant differences existed between the younger and older participants on this index (younger: 167 ± 135 ms, older: 128 ± 161 ms; two-tailed Student's *t*-test *t*(37) = −0.810, *P* = 0.423, *d* = 0.262). A lack of a difference is most consistent with the Inhibitory Deficit Theory, which would predict no differences in RT between age groups.

We also compared reaction times between correct and incorrect trials for the two age groups to determine the extent to which participants of different ages may have used different strategies between successful and unsuccessful trials. Participants could have used a strategy of making an indiscriminant first saccade and then apply the stimulus-response mapping to eventually gaze at the correct target location. In this scenario, participants would have faster reaction times on incorrect trials. Conversely, correct trials could represent trials with high confidence of the mapping, leading to faster reaction times on the correct trials. Neither the younger adults (correct: 715 ± 101 ms, incorrect 673 ± 129 ms; paired two-tailed *t*-test *t*(17) = 1.632, *P* = 0.121, *d* = 0.362), nor the older adults (correct: 902 ± 192 ms, incorrect 844 ± 169 ms; paired two-tailed *t*-test *t*(19) = 1.795, *P* = 0.086, *d* = 0.321) demonstrated a significant difference in reaction times between correct and incorrect trials, indicating participants simply chose the incorrect target on error trials without employing different cognitive strategies.

### 3.2. Visual Attention Domain

All three theories of cognitive aging would predict an age effect for visual attention errors, with older adults performing worse on that component of the task. However, we did not anticipate any condition effect in visual attention errors as we did not manipulate visual attention load across conditions. Participants had to identify the same six letters regardless of the number of saccade targets, while the six peripheral targets remained present at all times. Thus, we did not anticipate any effect of condition on the visual attention error rate, which was consistent with our results (*F*(1,37) = 0.225, *P* = 0.638, *h*_*p*_^2^ = 0.006). However, an effect of age was present. Older adults made 29% more errors in visual perception in the easy condition and 33% more errors in the hard condition than the younger adults (*F*(1,37) = 48.374, *P* < 0.001, *h*_*p*_^2^ = 0.567; Figure 2(d)) with no significant interaction present (age × condition interaction *F*(1,37) = 0.858, *P* = 0.360, *h*_*p*_^2^ = 0.023). This age difference was not due to differences in visual acuity as all participants had to correctly identify the letters prior to the start of the study. Rather, the temporal constraints placed on the identification of the letters made identification difficult for the older participants. The types of errors made by older adults were also consistent across the two task conditions. Older adults made 5.49% ± 8.16% false positives in the hard condition and 6.95% ± 10.26% false positives in the easy condition (compared to younger adults with false positive rate of 0.79% ± 1.66% in the hard condition and 1.94% ± 3.10% in the easy condition). Older adults missed cue letters 34.94% ± 18.01% of the time in the hard condition and 32.6% ± 14.01% of the time in the easy condition (compared to 6.73% ± 6.63% in the hard condition and 8.29 ± 9.32% in the easy condition for younger adults).

### 3.3. Relations among Measures of Attention

Although we first considered each dependent measure individually to assess the relative contributions of distractor suppression, processing speed, and correct target selection to performance on the cued saccade task in younger and older adults, we were also interested in how these measures related to one another. In younger adults, increasing the attentional load during a cued saccade task in either the visual or motor domain negatively impacted performance within that specific domain without affecting the opposite one, indicating a functional dissociation of the two processes in this age group [38]. We performed correlations among the dependent measures separately for the younger and older participants to identify potential relations between the measures and the extent to which these relations differed by age group (Table 2). Age positively correlated with visual attention errors (*r* = 0.519 and *P* = 0.019) and reaction time (*r* = 0.448 and *P* = 0.048) in the older participant group. In this group, reaction time positively correlated with visual attention errors (false positives and misses; Table 2, below the diagonal); thus older participants who were more accurate in identifying cue letters also had the shortest reaction times. Conversely, older participants with the shortest reaction times had the highest target selection error rates (Table 2, below the diagonal). Visual attention errors and target selection errors were negatively correlated. This was an interesting finding because, in younger adults, target selection errors and visual attention errors were unrelated (Table 2, above the diagonal). The younger adults did not show a strong relation between visual attention errors and reaction time as the older adults did, but younger participants did show a strong relation among motor attention measures. Young participants who made fewer initial saccade target selection errors were also more accurate in saccade endpoints (Table 2, above the diagonal). Also in this group, changes in target selection error rate positively correlated with reaction time. In sum, the relations among dependent measures varied greatly between younger and older performers and may shed some light on alternate strategies used by the different age groups.

## 4. Discussion

The present study evaluated visual and motor attention contributions to a cued saccade task, under temporal constraints, while varying motor attention load. Although the use of a cued saccade task to evaluate visual and motor attention is not new [44, 45], the approach of comparing and contrasting measures representing both visual and motor domains across age groups, to our knowledge, is novel. Typically, the visual component of the task covaries with motor components of the task, which may confound interpretation of results. In the present experiment, the visual attention load was maintained across both conditions so that any changes in performance could be attributed to changes in attention-mediated motor planning. We found that older adults demonstrated difficulty in both inhibiting irrelevant saccade targets and selecting correct saccade endpoints, but not slower processing, when the task was more complex.

### 4.1. Inefficient Selection of Task-Relevant, and Inhibition of Irrelevant, Motor Information by Older Adults

Behaviorally, it is oftentimes difficult to discern difficulties in selecting relevant information versus inhibiting irrelevant information. We had hypothesized that in the hard condition older participants would have proportionally greater difficulty in the motor component of the task than the younger participants (i.e., significant age by condition interaction), consistent with modality-specific difficulties in older adults [2, 36, 46] and in support of the Context Processing [26] and Inhibitory Deficit [29] Theories. This finding was observed in target selection errors and saccade endpoint accuracy. Few participants of any age made target selection errors in the easy condition in which one common saccade target was required for all six cue letters. However, older adults made significantly more incorrect initial saccades than the younger participants in the hard condition. This decrement in performance cannot be attributed to working memory errors or to difficulty in encoding task context, as older participants ultimately performed a saccade to the correct peripheral target after the initial incorrect saccade. However, this difficulty in initially selecting the correct saccade by the older adults could be attributed to difficulty in inhibiting saccades to incorrect targets [19, 29, 47]. Electrophysiological evidence in macaque has shown that as a decision to move to a particular location is made, activity coding the selected target increases and the activity related to the unselected movement decreases [48, 49]. This phenomenon has also been observed in the perceptual decision-making literature regarding saccade selection [50]. A number of authors have shown a parallel priming of motor responses during the sensory component of a task [51–53]. Presumably this preprocessing of movement contingencies allows for more efficient timing of movements. Difficulty in inhibiting undesired actions in older adults is robust, affecting arm reaching movements as well [37].

Changes in saccade endpoint accuracy between the easy and hard conditions also differed between young and old participants. We did not expect to find age-related differences in saccade endpoint accuracy in the easy condition, as older adults are as accurate as younger adults in simple saccade tasks [19, 25]. However, older adults had a significant decline in accuracy in the hard condition compared to the easy condition, and compared to the younger adults in the hard condition. Thus, when compared to younger adults, older adults have more difficulty with motor planning and execution when multiple movement options are available. Older adults may function better in less complex environments with fewer task components to attend [3, 4, 14].

### 4.2. Generalized Slowing in Older Adults

We had also predicted an age by condition effect in reaction time, which was not the case. Older adults were slower than younger adults in both conditions; however no interaction was present, a result consistent with others [34]. The presence of a generalized slowing of responses in older adults supports the Processing-Speed Theory of cognitive aging [20]. Correlations between age and reaction times for the older adults were 0.45 for both task conditions, similar to other reported relations between age and various speeded responses [54]. Assuming the easy condition represents a simple reaction time task (one response), the “cost” of having six choices for younger adults was not significantly different than the easy condition with one movement choice. Contrary to our results, Godefroy et al. [2] found a difference in “decisional cost” between younger and older adults between a simple and choice reaction time task. We may not have observed this effect due to the slower performance of the older adults on the easy condition. Older adults could have used two different strategies for the two conditions. They may have chosen accuracy over speed in the easy condition [55], yet chose speed over accuracy in the hard condition. In this case, reaction times for older adults might not significantly change between conditions as we found in the present study, yet target selection errors and saccade endpoint accuracy would be affected. The lack of difference in reaction times between the easy and hard conditions in the older adults may have also been due to the older adults not “preparing” for the upcoming saccade while waiting for the cue presentation in the easy condition. In this condition, the saccade was always to the same location regardless of the cue, so preparing the motor system for a saccade to the single target location would have been a useful strategy. Younger adults may have “preset” the motor system to make the single saccade and then focused on identifying when a cue target letter was shown, irrespective of which cue was shown. Parallel preparation of responses during the accumulation of sensory information occurs in young participants [51, 53, 56]. Older adults may have instead focused on perceiving the cue letter prior to considering the correct saccade direction, with a cost of longer reaction times. In tasks in which participants are correctly precued for the effector to use or future location of a stimulus most of the time, older adults typically do not “use” this precue information, leading at times to great accuracy than younger adults [35], but also to longer reaction times [2, 14, 17, 32, 35].

The increase in visual attention errors with age also supports the view of a general slowing of mental processing as a component of cognitive aging. We attribute the differences in visual perception errors between younger and older participants to difficulty in visual information processing rather than to declines in visual acuity. All participants were able to correctly identify target letters during training when no time constraints were imposed. Speed of visual perception has been shown to be similar between younger and older age groups based on evoked resting potentials [18]. Cerella [57] tested young and old participants on letter identification (4 possible letters) at various eccentricities and letter sizes. Older adults had less than a 10 ms delay in letter recognition with letters presented in the center of the screen, using letters slightly smaller than the ones used in the present study. For both age groups in that study, however, identification took longer than 250 ms. In our study, each target letter was only shown for 250 ms. In younger participants, a similar task with four cue letters can be performed with rapid serial visual presentation (RSVP) durations as low as 50 ms with less than 40% errors and in the same task at 125 ms RSVP durations with less than 10% visual perception errors [38]. We piloted the current experiment in three older adults at shorter RSVP times prior to data collection, and none of the participants were able to perform the task with letter presentation durations less than 250 ms. This need for slowing of the central RSVP may be indicating an inability to ignore other letters while attempting to identify the cue letter, or longer letter durations needed for information processing to identify the letter [2]. Older adults have more misses and false positives (both measures accounted for in our visual perception error rate) when performing a dual task when compared to younger participants [14], supporting the premise that older adults have difficulty inhibiting extraneous information.

### 4.3. Modality-Specific Changes Associated with Aging

Consistent with the notion of modality-specific slowing, previous studies have reported weak correlations between sensory and motor measures of performance in older adults [2, 36]. In the current study, we observed inverse or weak correlations between our visual and motor dependent measures. Older adults who performed best on the visual component of the task performed the worst on target selection for the initial saccade following cue letter presentation, which was the opposite for younger adults. Younger participants who performed best on the visual component of the task also performed the best on initial saccade target selection, although this relation was not significant. Two possibilities exist to explain these age differences. In younger adults, challenging one attentional system (visual or motor) has no effect on the other [38], indicating separate modality-specific attentional resources. However, in older adults, the inverse relation between performances on the visual component of the task versus target selection errors could be interpreted as evidence for shared attentional resources in this population. Older adults may allocate attentional resources differently to specific systems (e.g., visual, motor) as a compensation mechanism. Diminished attentional selection would negatively affect a person's ability to inhibit the noncue letters in the visual component of the task (temporal distractor suppression) and the incorrect targets in the motor component of the task.

A second possible explanation for the inverse relationship in the older adults may be due to slower processing speeds for selecting the correct visual cue and the correct application of the stimulus-response mapping. Temporal constraints of the task (250 ms duration of letter presentation in the RSVP) may have limited the overall time to complete a trial. This would have forced participants who spent more time to correctly perceive the cue letter to have to more rapidly select the correct saccade target, which may have led to more target selection errors. Conversely, other older participants may have made a more rapid decision on the presented cue letter, leading to more perceptual errors, but allowing for more time to select the correct target. In support of the second alternative, older adults had a positive correlation between visual perception errors and reaction time. Also, older participants who potentially took longer to select the visual cue, leading to greater visual attention errors, also had longer reaction times on correct trials.

## 5. Conclusions

The motor attention load-specific slowing in older adults suggests an attentional capacity issue related to the distribution of attentional resources across visual and motor modalities. In older adults, the consequences of increased motor planning complexity (e.g., increasing the number of possible saccade targets) are difficulty inhibiting unwanted movements and decreased eye movement accuracy. These changes may have an impact on an older adult's ability to function in a complex environment and may inform the development of effective techniques to maximize functional independence. Our results highlight the contributions of three different theories of cognitive aging on changes to attentional processes. Further study is required to fully assess the effect of potential differences in attentional capacity in younger and older adults on visuomotor tasks.

## Figures and Tables

**Table 1:** Means and standard deviations (in parentheses) for younger and older participants in the easy and hard cued saccade task conditions.

Dependent measure	Younger		Older	
	Easy	Hard	Easy	Hard
Visual attention error rate (%)	10.22 (3.36)	7.52 (3.61)	40.42 (3.52)	39.56 (3.27)
Target selection error rate (%)	0 (0)	30.72 (20.22)	1.84 (2.36)	44.69 (15.62)
Reaction time (ms)	568.50 (78.42)	735.79 (132.91)	773.79 (142.03)	902.33 (191.17)
Saccade endpoint accuracy (°)	2.58 (1.08)	2.86 (1.07)	2.81 (0.934)	3.41 (0.882)
Saccade endpoint variability (°)	1.69 (1.00)	1.79 (0.799)	1.60 (0.586)	1.78 (0.715)

**Table 2:** Relations among measures of attention for younger and older participants.

	VAE	VAE	VAE	VAE
VAE	—	0.426	0.041	−0.172
TSE	−0.452*	—	0.481*	−0.456*
SEA	0.165	0.229	—	−0.325
RT	0.577**	−0.625**	−0.194	—

*Note*. Correlations for younger participants (*n* = 19) are presented above the diagonal, and correlations for older participants (*n* = 20) are presented below the diagonal (VAE: visual attention errors (misses and false positives); TSE: target selection errors; SEA: saccade endpoint accuracy; and RT: reaction time).

*Correlation is significant at *P* < 0.05 (2-tailed).

**Correlation is significant at *P* < 0.01 (2-tailed).
